# Emerging Prospects for Nanoparticle-Enabled Cancer Immunotherapy

**DOI:** 10.1155/2020/9624532

**Published:** 2020-01-03

**Authors:** Manal Ali Buabeid, El-Shaimaa A. Arafa, Ghulam Murtaza

**Affiliations:** ^1^Department of Clinical Sciences, College of Pharmacy and Health Sciences, Ajman University, Ajman 346, UAE; ^2^Department of Pharmacy, COMSATS University Islamabad, Lahore Campus 54000, Pakistan

## Abstract

One of the standards for cancer treatment is cancer immunotherapy which treats both primary and metastasized tumors. Although cancer immunotherapeutics show better outcomes as compared with conventional approaches of cancer treatment, the currently used cancer immunotherapeutics have limited application in delivering cancer antigens to immune cells. Conversely, in solid tumors, tumor microenvironment suppresses the immune system leading to the evasion of anticancer immunity. Some promising attempts have been made to overcome these drawbacks by using different approaches, for instance, the use of biomaterial-based nanoparticles. Accordingly, various studies involving the application of nanoparticles in cancer immunotherapy have been discussed in this review article. This review not only describes the modes of cancer immunotherapy to reveal the importance of nanoparticles in this modality but also narrates nanoparticle-mediated delivery of cancer antigens and therapeutic supplements. Moreover, the impact of nanoparticles on the immunosuppressive behavior of tumor environment has been discussed. The last part of this review deals with cancer immunotherapy using a combination of traditional interventional oncology approach and image-guided local immunotherapy against cancer. According to recent studies, cancer therapy can potentially be improved through nanoparticle-based immunotherapy. In addition, drawbacks associated with the currently used cancer immunotherapeutics can be fixed by using nanoparticles.

## 1. Introduction

**Figure 1:**
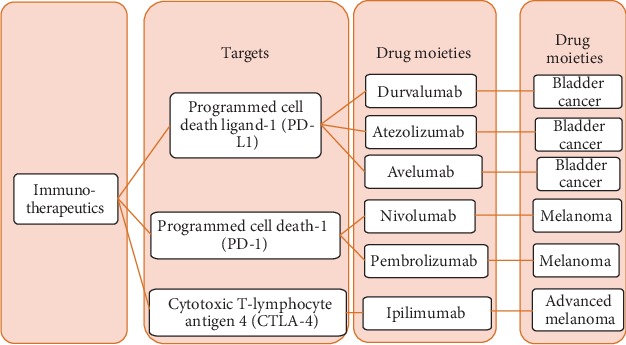
Examples of immunotherapeutics (mainly monoclonal antibodies) approved by the FDA for cancer treatment.

Cancer is one of the most lethal diseases and is causing thousands of deaths annually throughout the world [1]. It is traditionally treated by using anticancer medicines and radiations [2]. However, these modalities are associated with certain drawbacks such as the high possibility of recurrence, limited therapeutic effectiveness, and distressing undesired effects. In recent years, clinicians have promisingly treated cancer by using immunotherapeutic moieties [3]. This approach has several advantages such as its effectiveness against metastasized cancer also as well as low risk of recurrence [4, 5]. Owing to these features, clinicians are interested in opting immunotherapy as a standard treatment option against cancer [6]. Thus, the researchers are actively developing different immunotherapeutic antibodies [6] and cell therapeutics [7]. Particularly, antibodies have been used in the development of immune checkpoint inhibitors against various regulatory molecules/receptors (Figure 1). Nonetheless, some undesired effects are also associated with cancer immunotherapeutics such as autoimmune disease [3]. In addition, immunotherapeutics are more effective against lymphoma than solid tumors [8, 9] likely due to difficult penetration of immunotherapeutic agents through their abnormal ECM (extracellular matrix) [10, 11]. Moreover, immune-suppressive tumor microenvironment (ISTM) is also responsible for the reduced efficacy of immunotherapeutics against solid tumors [12, 13].

Current research work is focused on the management of cancer immunotherapeutics' shortcomings, for instance, by using nanoparticles [14]. Nanoparticles are the biomaterial-based nanosized vehicles [15, 16] which are extensively used in delivering drug molecules in a controlled fashion as well as to the target site [17].

Cancer treatment using immunotherapeutics depends on three important factors. The first factor deals with an effective transfer of cancer antigens to immune cells, particularly APCs (antigen-presenting cells), such as dendritic cells. The induction of anticancer immune response after delivery of adjuvant and cancer antigen to immune cells is the second requirement for this treatment. The third factor involves the modulation of the IDTM to induce a response to the anticancer immunotherapeutics. These aims can be achieved by using nanoparticulate systems, which can be potentially utilized for the induction of immune response against cancer. This review article describes the current trends in cancer therapy using nanoparticles as immune-modifying systems.

## 2. Mode of Action of Immunotherapeutics in Cancer

**Figure 2:**
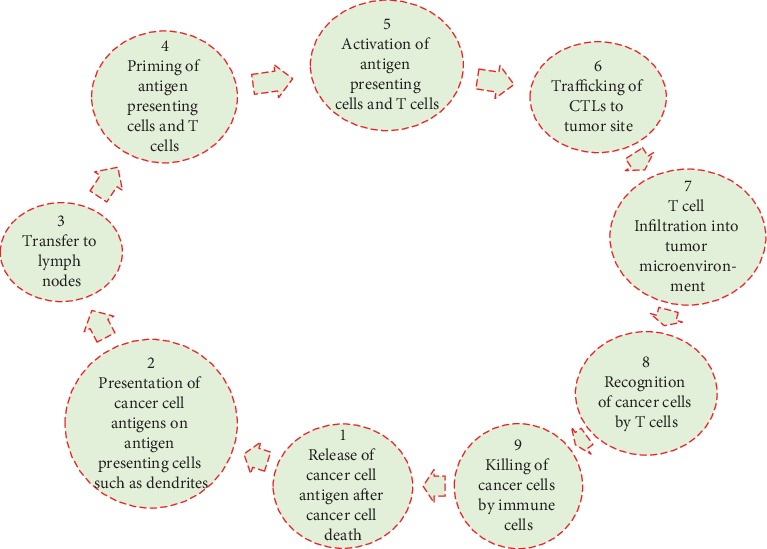
Cancer-immunity cycle showing its main stages such as release, presentation, transfer, priming, activation, trafficking, infiltration, recognition, and killing.

For the application of nanoparticles in the treatment of cancer, it is a prerequisite to comprehend the mechanistic aspects of cancer immunotherapy. The framework of cancer immunotherapy research depends on a cancer-immunity cycle (Figure 2) which involves the removal of tumor cells. Necrosis- or apoptosis-mediated death of cancer cells produces tumor antigens. APCs capture these antigens and present on major histocompatibility complex (MHC). The complexity of dendritic cells and cancer antigens induces the priming of immature T cells in the lymph nodes, followed by the infiltration of the activated TCLs (tumor-specific cytotoxic T lymphocytes) into the tumor site. TCLs interact with T cell receptors and MHC to recognize tumor cells. Then, effector T cell-mediated apoptosis of cancer cells releases additional cancer antigens which strengthen the immune response. These events lead to the induction of effective immunity against cancer, which is, however, interrupted by several barriers.

Proinflammatory cells, for instance, M1-polarized macrophages possess the capability of killing tumor cells. The deceased cells produce various immunosuppressive factors such as IL-10 (interleukin-10) inducing repolarization of macrophages from M1 to M2 [18–20]. In addition, these dead cells release the characteristic substances (for instance, monocyte chemoattractant protein-1 or MCP-1) which attract various cells (for example, leukocytes) towards them [19, 20], leading to the transfer of monocytes and MDSCs (myeloid-derived suppressor cells) into the tumor microenvironment [21–23]. Here, the differentiation of these monocytes into TAMs (tumor-associated macrophages) takes place. TAMs accelerate the growth of the tumor and camouflage it from immune attack [19–23]. On the other hand, the infiltrated MDSCs play a role in the inhibition of immune response against cancer through the secretion of anti-inflammatory cytokines, leading to Treg cell activation. Treg (regulatory T) cells have an immunosuppressive function and inhibit the maturation process of dendrites, resulting in the remission of the tumor [24–26]. The situation becomes more problematic when tumor evasion from an anticancer immune effect occurs due to the inhibition of TCLs by immune-suppressive entities present on PD-1, PD-L1, PD-L2, and CTLA-4 cells. Eventually, these phenomena limit the immunotherapeutic efficacy [27–30], revealing the significance of solving the issues of current immunotherapies against cancer. Immunotherapy can be intervened by nanomaterials to enhance immunity against cancer.

## 3. Types of Nanoparticle Systems

**Figure 3:**
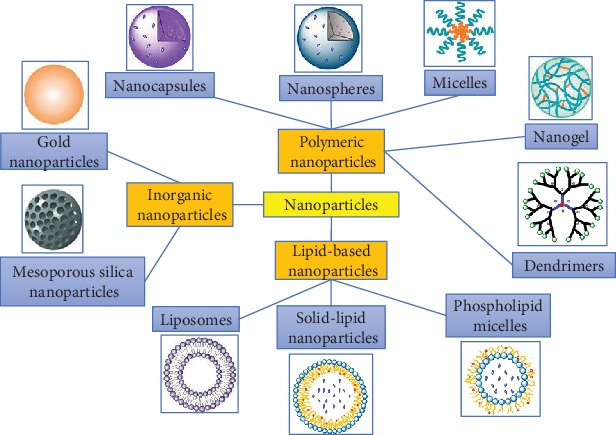
The representative examples of currently studied nanoparticles (polymeric, lipidic, and inorganic) for cancer immunotherapy.

During current years, several nanoparticle systems (Figure 3) have been studied for cancer immunotherapy. Among a wide array of the currently studied nanoparticles for cancer immunotherapy, polymer-based nanoparticles are the most popular systems [31]. The Food and Drug Administration (FDA) has approved a variety of polymers, such as polyethylene glycol, poly (lactide-o-glycolic) acid, and chitosan owing to their biodegradable, biocompatible, and nontoxic features, for the synthesis of nanoparticle systems for cancer immunotherapy [32]. Other commonly used nanoparticulate systems include the inorganic (such as gold nanoparticles) and the lipid-based nanoparticles (such as liposomes) [33], as mentioned in Figure 3. All of these nanoparticles can be promisingly used for targeting cancer and delivering antigens and supplements to the target site with a good accuracy and precision for the activation of the immune system.

## 4. Current Strategies for the Preparation of Nanoparticle Systems

Nanoparticles are produced through physical, biological, and chemical methods. Biological methods are mainly used for microorganism-assisted biogenesis of metallic nanoparticles such as gold nanoparticles [34]. Several approaches including emulsification, sol-gel synthesis, precipitation, spray drying, and salting out. Nanoemulsification is the generally adopted technique for the fabrication of polymer nanoparticles. This process involves the removal of organic solvents by the process of evaporation or extraction, leaving polymer nanoparticles in the pot [35]. However, it is crucial to remember that the selected approach affects the properties of the acquired nanoparticles, including size, shape, and charges [35].

## 5. Optimum Features of Nanoparticles for Efficient Immunotherapy

Nanoparticles possess distinguished physicochemical properties including size, shape, and charge, which can be customized to achieve various therapeutic goals such as cancer immunotherapy [36]. For example, the size of nanoparticles affects the cellular uptake and endocytosis. As compared with the larger nanoparticles (>100 nm), smaller ones (25-40 nm) have a greater potential of immune response activation, since smaller nanoparticles are allowed to move to lymph nodes via dendritic cells, while the larger ones are retained at the target site. Very large nanoparticles (>500 nm) are engulfed in the macrophages through phagocytosis [37]. In addition, the nanoparticle's shape also influences its uptake and distribution. Nonspherical nanoparticles experience prolonged systemic circulation, because of their potential to avoid nonspecific cellular phagocytosis. On the other hand, nonspherical nanoparticles are more readily engulfed by dendrites, in comparison with spherical nanoparticles [38]. Furthermore, the surface charge of nanoparticles also influences the mechanism of their internalization. For instance, cationic nanoparticles are quickly engulfed by macrophages or dendrites, resulting in a significant lysosomal escape. Conversely, there is stronger affinity between cationic nanoparticles and serum proteins, which provokes the reaction of cationic nanoparticles with anionic components such as hyaluronic acid and other moieties in the tumor microenvironment, resulting in the reduced leakage of nanoparticles from tumor tissues. In addition, charged nanoparticles have a lesser penetration depth and shorter circulation time than that of neutral nanoparticles [39]. Moreover, tumor-targeting antibodies can be conjugated to the nanoparticles to achieve the enhanced permeability and retention effect (EPR) [36].

## 6. Multifunctional Nanoparticle Systems

**Figure 4:**
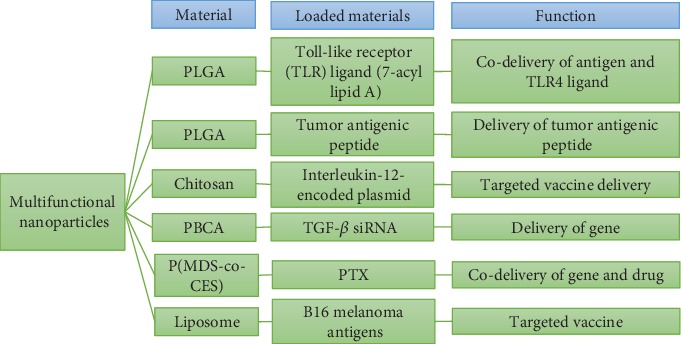
Representative examples of multifunctional nanoparticulate systems studied *in vivo* for cancer immunotherapy [43–50]: PLGA-Poly(lactide-o-glycolic acid), PBCA-Polybutyl cyanoacrylate, and P(MDS-co-CES)-A triblock polymer.

A considerable development in the field of cancer immunotherapy has been introduced during the last few years. However, clinical trials of cancer vaccines could not receive significant success. In addition to several other factors, this unremarkable accomplishment could be due to the fact that traditional methods of drug delivery techniques were not safe. In recent years, new opportunities, especially nanoparticle-based modalities, have been explored for the treatment of cancer [40]. Particularly, cancer vaccines have been promisingly delivered using multifunctional nanoparticles, which exhibit several benefits, including targeted delivery of immunotherapeutics (such as immune checkpoint inhibitors) using stimuli-sensitive materials resulting in the reduced off-target effects and increase in drug efficacy. Other advantages of nanoparticle system is the simultaneous delivery of multiple therapeutic moieties, where treatment and imaging agents can be integrated in the core and on the surface of multifunctional nanoparticles for cancer targeting [41]. Some representative examples of multifunctional nanoparticulate systems for cancer immunotherapy are presented in Figure 4. Current studies have revealed that nanoparticles have multifaceted functions for (a) working as an effective substitute for generation and transduction of CAR- (chimeric antigen receptor-) T cell, (b) inculcating tumor-suppressing activity to TAM (tumor-associated macrophages), and (c) knocking down Kras oncogene addition by using nano-Crisper-Cas9 delivery system [42]. In addition, nanomedicine platform can be repurposed for the improvement of cancer therapy function by using multifunctional nanoparticles.

## 7. Nanoparticle-Mediated Delivery of Tumor Antigens

The induction of tumor immunity requires the effective transfer of tumor antigens to APCs. The researchers have introduced two important classes of antigens, i.e., TAAs (tumor-associated antigens) and TSAs (tumor-specific antigens, also called neoantigens). Although TAAs are mainly expressed on cancer cells, normal and differentiating cancer cells also contain TAA contents. Thus, an autoimmune reaction might be caused when these antigens are used as immunotherapeutic targets. Alternatively, the autoimmune problem is not observed in the case of TSAs, since they are expressed in cancer cells only. However, the human enzyme system easily degrades these innate tumor antigens. In addition, these antigens are less efficiently transferred to immune cells; thus, they are known as weak immunogenic species. Since secondary lymphoid organs primarily home the immune response, an effective anticancer immune response can only be initiated when the lymph nodes are efficiently accessed by tumor antigens. In view of that, a nanoparticle-mediated safe delivery of tumor antigens to lymph nodes has been extensively investigated [51]. These studies have revealed two main benefits, i.e., tumor antigen protection against biodegradation and their targeted delivery to the lymph nodes. Afterward, successfully and safely delivered nanoparticles undergo an effective internalization into APCs [52]. Most of the abovementioned problems have been solved by using nanoparticles for the delivery of tumor antigens. However, the synthesis and use of the nanoparticles for this purpose are required to comply with many considerations.

Nanoparticle delivery to lymph nodes is delicately affected by several factors such as water solubility, shape, size, and surface charge of nanoparticles [53–56].Hydrophobic polymers (for instance, chitosan) or polymers having hydrophobic component exhibit intrinsic adjuvant activity and show potential to activate immune cells even in the absence of additional signals [56, 57]. For example, the increase in side chain lipophilicity of PGA (poly(gamma-glutamic acid)) nanoparticles results in ameliorated uptake of antigen, increased activation of dendrites, and improved cellular response [58].

In addition to size, particle shape also affects nanoparticle drainage from lymph nodes. Nanoparticles have been prepared in a variety of shapes such as spherical, discs, rods, and stars [58]. However, spherical nanoparticles have better properties than other shapes in respect of migration effect, infiltration capacity, and circulation time [59–61].

Furthermore, transportation of antigen-loaded nanoparticles depends on their size. Nanoparticle size neither should be lesser than 5 nm (termed as small-size nanoparticles) to prevent their leakage from the circulatory system nor greater than 100 nm (termed as large-size nanoparticles) to avoid their entrapment in ECM and lymph nodes. Nanoparticles having a size of approximately 5–100 nm (termed as medium size nanoparticles) exhibit a prolonged circulation time and can be used to target the lymphatic system. For instance, PPS (poly(propylene sulfide)) nanoparticles having a size range of 20-45 nm persisted in the lymphatic system for about five days [57]. Additionally, APCs, lymph nodes, and dendritic cells contained almost half of these nanoparticles [15, 57]. A study on the comparison of nanoparticles having a size of 25 nm (smaller) and those with 100 nm (larger) after intradermal administration reported more efficient delivery of smaller nanoparticles to lymph nodes through the lymphatic system [15, 55]. Nonetheless, the optimum size of antigen-loaded nanoparticles for efficient delivery to lymph nodes is 5–100 nm. These nanoparticles can be chemically modified via attaching suitable ligands such as mannose for their active transport to the lymph nodes.

Furthermore, the nanoparticle surface charge not only influences the cellular internalization but also affects the immune response activation [62]. In general, positively charged nanoparticles exhibit a higher immune response but a lower tissue permeability than the negatively charged or inert ones. The reduced permeability could be attributed to their immobilization in the oppositely charged ECM [63]. As compared with the negatively charged or inert nanoparticles, positively charged nanoparticles are easily taken up by the dendritic cells localized at the site of injection. On the other hand, hemolysis and platelet aggregation and thus the premature antigen release are the critical problems associated with lymphatic transport of cationic nanoparticles [64, 65].

## 8. Nanoparticle-Mediated Delivery of Therapeutic Supplements

Therapeutic supplements (TS), also known as adjuvants, are used in combination with tumor antigens to enhance their mutagenicity. TS have a resemblance to pathogenic molecules which are identified by pattern recognition receptors (PRRs) [66–69]. An example of TS used in cancer immunotherapy is lipopolysaccharide. The internalization of TS with tumor antigens into APCs results in an ameliorated immune response against cancer through the induction of a strong antigen-specific T cell response [70–73]. In addition, the combination of nanoparticle-mediated delivery of tumor antigen with immune checkpoint blockade improves the immune response against cancer. Therefore, different types of solid tumor and blood cancer can be potentially treated by using nanoparticulate systems.

**Figure 5:**
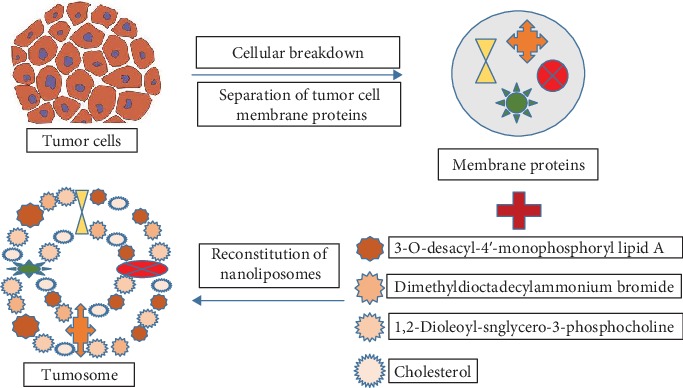
Diagrammatic presentation of multifaceted tumosome for cancer immunotherapy.

A recent study described the simultaneous delivery of tumor-specific antigens (TSAs) and TS using nanoliposomes (Figure 5) having a multifaceted immunomodulatory effect [74]. Nanoliposome size was reported as 100 nm and denoted by the term “tumosomes”. It contained two immunostimulatory TS, i.e., MPLA (3-O-desacyl-4′-monophosphoryl lipid A) and DDA (dimethyldioctadecylammonium) as a danger signal and a cell-invasion domain, respectively. The findings revealed an enhanced anticancer immunity, reduction in tumor growth, and improved survival of mouse tumor models treated with the tumosomes. In this approach, self-antigens may face a condition of autoimmunity that could be overcomed by using TSAs. In addition, the therapeutic efficacy of this modality can be further improved by using it with other therapeutic approaches including chemotherapy.

## 9. Nanoparticle-Mediated Delivery of Immunomodulators

Tumors can create immunosuppressive tumor microenvironment which can enhance cancer growth and metastasis. Thus, cancer can be potentially treated by immunomodulation of tumor microenvironment [75].

**Figure 6:**
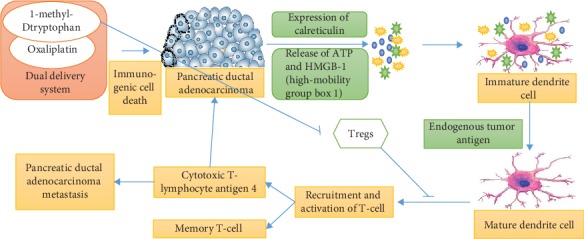
Diagrammatic presentation to express the combined effect of 1-methyl-D-tryptophan and oxaliplatin on immune response in pancreatic ductal adenocarcinoma. A vehicle was prepared for the codelivery of two chemotherapeutics, i.e., 1-methyl-D-tryptophan and oxaliplatin. 1-Methyl-D-tryptophan plays a role in causing immunogenic cell death via expression of calreticulin and release of ATP and HMGB-1 (high-mobility group box 1), while the interference of oxaliplatin with the indoleamine 2,3-dioxygenase 1 pathway is reported. After receiving adjuvant stimuli and uptaking the dying tumor cells, dendrite cells undergo a maturation process along with crosspresentation of tumor antigens. Afterward, primary and metastasized cancer cells are killed by CD+ T cells through granulysin and perforin. This codelivery system influences the indoleamine 2,3-dioxygenase 1 pathway, interferes Treg development, and controls other immunomodulatory activities, resulting in the strengthening of the apoptotic effect by the immune system. The immunogenic cell death pathway involves the activation memory T cells and helper cells which helps in the prevention of disease recurrence.

One of the potential examples of immunosuppressive T cells is Tregs (Figure 6) which can suppress the activity of anticancer T-effector cells. Tregs are involved in the prevention of autoimmune disease via the establishment of immune tolerance against autoantigens. However, in cancer, Tregs can exert a suppressive effect on immune cells in the tumor microenvironment resulting in the reduced anticancer immunity. Antitumor immunity can be induced by inhibition of elimination of Tregs [76]. For instance, anti-CTLA-4 is a checkpoint blockade that is utilized for the control of Tregs' activity in cancer immunotherapy. Moreover, Tregs can be removed from the tumor microenvironment by the engineering of Treg-targeted nanoparticles [77].

Tumor microenvironment contains a high level of TAMs. These are the immune cells which generate an excess of immunoregulatory cytokines such as TGF- (transforming growth factor-) *β* and IL-10. In addition, TAMs produce inflammatory cytokines such as IL-6 leading to the suppression of anticancer immune responses. Thus, effective cancer immunotherapy requires targeting and killing TAMs in the tumor microenvironment utilizing surface-modified nanoparticles.

Hepatic, lung, and breast cancer exhibit overexpression of various cytokines including TGF-*β* which suppresses activation, maturation, and differentiation of immune cells. Therefore, an immune response in cancer might be induced through the suppression of the TGF-*β* in the tumor microenvironment. In a recent study, nanoparticles were prepared by the process of microencapsulation for the delivery of TGF-*β* inhibitors to the tumor microenvironment. It resulted in the induction of both innate and adaptive immune activities leading to the inhibition of tumor growth as well as an improvement in the survival of mice having metastatic melanoma.

Tumor microenvironment of hepatic, gastrointestinal, and breast cancer contains high levels of tumor-suppressor cells such as MDSCs which generate various cytokines such as IL-10 for the activation of Tregs and inhibition of other immune cells. In this context, effective cancer immunotherapy requires MDSC elimination in the tumor microenvironment. Nanoparticle-mediated delivery of immunomodulators to the tumor microenvironment can be accomplished via active or passive transport. Thus, the ameliorated anticancer immune effect and the reduced undesired effects can be acquired through nanoparticle-mediated delivery of immunomodulators to the tumor microenvironment.

The recent studies combined various therapeutic approaches (such as checkpoint blockade immunotherapy and nanoscale metal-organic structure-aided radiotherapy) with nanotechnology to overcome the immunosuppressant microenvironment of tumor-facilitating effective treatment of tumor [78–82]. The researchers are very optimistic to overcome the drawbacks of currently used cancer immunotherapy by utilizing these combined modalities.

## 10. Localized Anticancer Immunotherapy

The hundreds of studies have reported the synthesis of nanoparticles for the treatment of cancer; however, the majority of the developed nanoparticulate systems could not be translated into clinical use. A review published in 2016 on the nanoparticle-based studies conducted during the last 10 years revealed the delivery of <1% of the intravenously administered dose to solid tumors [83]. It could be due to the tumor microenvironment which comprises heterogeneous structure and the distorted vasculature system, resists the entrance of drug molecules into the tumor site, and thus suppresses antitumor efficacy. In this context, novel nanoparticulate systems have been developed for local administration which have greatly attracted the attention of cancer clinicians [84].

Interventional radiology is a branch of interventional oncology which deals with the use of image guidance for the localized diagnosis and treatment of cancer using a minimum surgical procedure [85]. Anticancer therapeutics can be delivered to various malignant areas using medical imaging technology, for instance, conjunction of MRI (magnetic resonance imaging) and catheters. The image guidance approach can be used in local therapy to achieve various benefits such as reduced dose, cost-effectiveness, lesser undesired effects, and swift response [86].

Nanoparticles have several versatile features which pave their use in the fabrication of various imaging agents. For instance, ferric oxide nanoparticles [87, 88] and gold nanoparticles [89] are widely used as contrast agents in MRI and CT scan, respectively. Consequently, such functional nanoparticles can be utilized in the development of injectable medicines for their local use in medical imaging.

The currently available anticancer immunotherapeutic agents are directly administered to the circulatory system of the patients which leads to low efficacy and high toxicity. For instance, a high dose of an immune checkpoint suppressor is required when it is administered as an intravenous infusion. However, a stronger anticancer T cell activity with a low risk of side effects can be induced through local administration of an immune checkpoint suppressor, even at low doses [90, 91]. Nonetheless, the efficacy of cancer immunotherapeutics can be improved while its associated side effects can be reduced through local immunomodulation [92]. Even, the systemic anticancer immunity can be promoted by activation of the locally injected immune cells. In addition, the situation in which systemic infusion is associated with the production of large amounts of serum antibodies can be avoided by using local immunotherapy. It leads to the reduced activity of nonspecific immune cells, diminished side effects, and suppressed inflammatory processes [93].

Thus, the locally administered nanoparticles which have imaging characteristics and can exert effective immunotherapeutic effect against cancer have gained promising importance. Nanoparticles loaded with low-dose immunotherapeutics can be developed by combining traditional interventional oncology approach with image-guided local immunotherapy against cancer to safely target immunological organs or solid tumors. One of the important features of this modality is the use of imaging devices for the confirmation of immunotherapeutic delivery to the target area.

In image-guided local immunotherapy, the disposition of immunomodulatory agents can be monitored by imaging the nanoparticles loaded with cancer immunotherapeutics such as cancer antigens, cytokines, and adoptive cell therapeutic moieties. Consequently, conventional anticancer therapies can be replaced with more efficacious cancer therapy comprising cancer immunotherapy, nanotechnology, and interventional oncology.

## 11. Conclusion

The current research has revealed the application of biomaterial-based nanoparticles in the amelioration of anticancer immunity. Nanoparticles can improve antigen presentation via efficient delivery of cancer antigens and therapeutic supplements to APCs in immunological organs, for example, lymph nodes. Therefore, a vaccine-like prolonged and broader immune effect can be yielded by utilizing nanoparticle-loaded cancer immunotherapeutics as compared with free immunotherapeutic agents. For instance, neoantigens based on mRNA (mRNA-nAg) are less immunogenic but its translation in the cytoplasm can enhance T cell activity [94]. However, ubiquitous nucleases can degrade such agents and hinder their delivery into APCs. It is a valuable approach to deliver mRNA-nAg to immune cells by using nanoparticles [95]. Furthermore, nanoparticle-mediated delivery of immunomodulators to the tumor microenvironment can initiate the process of immune surveillance [41]. Such drugs can be efficiently delivered to the tumor site by using characteristic nanoparticles which respond to the tumor microenvironment. Furthermore, nanoparticulate systems can be combined with other modalities such as radiotherapy [96], chemotherapy [97], and phototherapy [98, 99] to improve the therapeutic efficacy of cancer immunotherapy. A few years back, for nanoparticle-loaded cancer immunotherapeutics, systemic administration was the preferred route of administration which caused toxic effects because of high doses. A few years back, nanoparticle was used to deliver cancer immunotherapeutics into systemic circulation; however, it required high doses of immunotherapeutics which caused toxic effects. Therefore, a new modality, named as image-guided local immunotherapy, is developed by combining the traditional interventional oncology approach with local cancer immunotherapy. This new modality produces therapeutic effectiveness even at low doses of immunotherapeutics due to their site-specific delivery and thus is associated with reduced toxicity [100]. In addition, immune cells or antibodies can be mimicked by using the synthesized nanoparticle based on the advance knowledge of mechanisms involved in cancer immunity. Recent advancement in the field of cancer immunotherapeutics is the development of nanoparticle-based artificial APCs [101], which can be used instead of natural APCs for the activation of the adaptive anticancer immune response.

The abovestated literature reveals that the interdisciplinary research, especially the union of various biomedical approaches, has evolved into current cancer immunotherapy. However, the development of biomaterial-based anticancer immunotherapy requires a detailed knowledge of how biomaterials interact with the immune system. For cancer immunotherapy, nanoparticle development using biomaterials has played an important role in achieving therapeutic efficacy at comparatively low doses and avoiding toxicity. In short, cancer patient's life quality and span can be improved by developing cancer vaccines based on nanoparticles.
